# NRF2 transcriptionally regulates Caspase-11 expression to activate HMGB1 release by Autophagy-deficient hepatocytes

**DOI:** 10.1038/s41420-023-01495-x

**Published:** 2023-07-28

**Authors:** Bilon Khambu, Genxiang Cai, Gang Liu, Niani Tiaye Bailey, Arissa A. Mercer, Kamal Baral, Michelle Ma, Xiaoyun Chen, Yu Li, Xiao-Ming Yin

**Affiliations:** 1 Department of Pathology & Laboratory Medicine, Tulane University School of Medicine, New Orleans, LO USA; 2 Department of Pathology & Laboratory Medicine, Indiana University School of Medicine, Indianapolis, IN USA; 3 CAS Key Laboratory of Nutrition, Metabolism and Food Safety; Shanghai Institute for Biological Sciences, Chinese Academy of Sciences, Shanghai, China

**Keywords:** Inflammasome, Macroautophagy

## Abstract

Injury or stress can induce intracellular translocation and release of nuclear HMGB1, a DAMP molecule known to participate in inflammation and other pathological processes. Active release of HMGB1 from stimulated macrophages can be mediated by inflammasomes, which cleave Gasdermin D to form pores on cytoplasmic membranes. We previously had shown that active release of HMGB1 from autophagy deficient hepatocytes also depended on the inflammasome but how the inflammasome was activated was not known. Here we report that persistent activation of transcription factor NRF2 under the autophagy deficient condition led to transcriptional upregulation of *Caspase-11* expression, which could then activate the CASPASE-1inflammasome. Using chromatin immunoprecipitation (CHIP) and luciferase-based reporter assays, we show that NRF2 directly binds to the *Caspase-11* promoter and transcriptionally increase the expression of *Caspase-11*. Genetic deletion of *Caspase-11* in autophagy-deficient livers represses the release of HMGB1 and its pathological consequence, ductular cell proliferation. Consistently, deletion of NLRP3, which can activate CASPASE-1 mediated inflammasomes under other types of signals, did not prevent HMGB1 release and ductular cell proliferation in autophagy deficient livers. Surprisingly, while cleavage of GASDEMIN D occurred in autophagy-deficient livers its deletion did not prevent the HMGB1 release, suggesting that CASPASE-11-mediated inflammasome activation may also engage in a different mechanism for HMGB1 release by the autophagy deficient hepatocytes. Collectively, this work reveals the novel role of NRF2 in transcriptional upregulation of *Caspase-11* and in inflammasome activation to promote active release of HMGB via a non-Gasdermin D mediated avenue.

## Introduction

High-mobility group box 1 (HMGB1) is a danger-associated molecular pattern (DAMP) molecule that is expressed virtually in all eukaryotic cells. Besides its nuclear chromatin chaperone function, HMGB1 can be actively secreted or passively released from severely stressed or injured cells, respectively [1–5]. Extracellular HMGB1 interacts with various binding partners such as receptor for the advanced glycation end product (RAGE), and Toll-like receptor 4 (TLR4) to trigger inflammation following infection or injury [5]. While active HMGB1 release is best studied in LPS-stimulated macrophages, epithelial cells such as hepatocytes can also actively release HMGB1 under various conditions [3, 6].

Autophagy is an intracellular lysosomal degradative pathway that is vital for hepatic homeostasis. Disruption of autophagy results in hepatic inflammation, fibrosis, proliferation of hepatic progenitor cells (HPCs) or ductular cells (DCs), and tumor development [3, 7]. These pathological changes reflect the different stages of common liver diseases. We have reported in previous studies that autophagy deficiency in hepatocytes induces intracellular translocation and active release of HMGB1 [3]. HMGB1 release under this condition does not impact hepatic inflammation or fibrosis but expands DCs and promotes hepatic tumorigenesis [3].

HMGB1 is considered as the “leaderless protein” as it lacks the conventional N-terminal hydrophobic secretion signal peptide [5, 8], which suggests that HMGB1 cannot be secreted by the conventional ER to Golgi-dependent secretory pathway. Studies have shown that HMGB1, like other leaderless proteins IL-1β and IL-18, can be actively released from LPS-stimulated macrophages via the cytoplasmic pore formed by GASDERMIN D after its cleavage by CASPASE-1 and/or CASPASE-11 [9, 10]. We have also shown that the inflammasome is involved in the active release of HMGB1 from autophagy-deficient hepatocytes [3]. However, how the inflammasome in the autophagy deficient hepatocytes was activated and the role of GASDERMIN D were not known.

Here, we report that CASPASE-11 is required in the active release of HMGB1 from the autophagy-deficient hepatocytes. *Caspase-11* is transcriptionally upregulated by a novel mechanism involving the transcription factor NRF2, which is elevated in autophagy-deficient condition. Surprisingly, GASDERMIN D, a critical component required for HMGB1 release by the macrophages, is dispensable for HMGB1 release by the autophagy deficient hepatocytes. These results indicate a novel mechanism of HMGB1 release in hepatocytes, which is dependent on NRF2 but not on GASDERMIN D.

## Results

### CASPASE-11 activation is involved in HMGB1 release and ductular reaction in autophagy-deficient livers

We have previously demonstrated that autophagy-deficient hepatocytes release HMGB1, which requires activation of inflammasome [3]. CASPASE-1 is the executor of the canonical inflammasome and activates downstream targets such as IL-1β and IL-18, through proteolytic cleavage [11]. Another caspase known as CASPASE-11 in mouse and CASPASE-4 in human can also be the executor in the non-canonical inflammasome [12]. Moreover, CASPASE-11 physically interacts with CASPASE-1 and can contributes importantly to the activation of CASPASE-1 inflammasome [13]. Additionally, the inflammasome is also required for the activated macrophages to release the cleaved IL-1β, and IL-18, and intact HMGB1 to extracellular space through the cytoplasmic pores formed by GASDERMIN D, whose cleavage by CASPASE-1 and/or CASPASE-11 is necessary for this step [12, 14].

We had reported that both CASPASE-1 and CASPASE-11 were activated in the autophagy-deficient livers that lack a key autophagy gene, Atg7 [3]. Using the commercially available *Caspase-1* deficient mice (The Jackson Laboratory Cat No 016621), which also carry a naturally occurring *Caspase-11* mutation that eliminates its function we found that the inflammasome was required for the release of HMGB1 from autophagy-deficient hepatocytes and its subsequent pathological effect in ductular cell proliferation [13]. It is, however, unclear whether CASPASE-1 or CASPASE-11 or both plays important role in HMGB1 release and ductular reaction in the autophagy-deficient liver. Mice with *Caspase-11* only deletion, but not *Caspase-1* only deletion, are available commercially and provide the opportunity to determine the direct role of CASPASE-11 in HMGB1 release by hepatocytes.

**Fig. 1:**
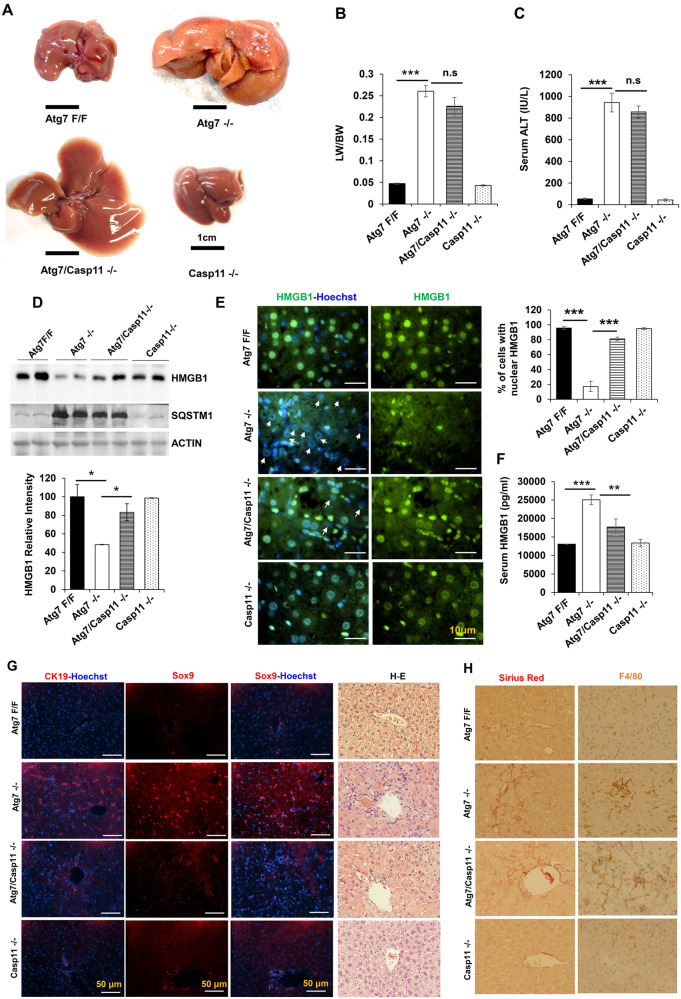
*Caspase-11* deletion prevents HMGB1 release and ductular cell proliferation in the autophagy-deficient liver. **A** Representative gross morphology of the livers of 9-week-old mice of the indicated genotypes. **B**, **C** The liver weight and body weight (LW/BW) ratio and the serum ALT levels were determined for 9-week-old mice. **D** Total liver lysates from 9-week-old mice were analyzed by immunoblotting. Protein band intensity was normalized to ACTIN band intensity. **E** Liver sections were stained for HMGB1. Arrows indicate hepatocytes without nuclear HMGB1.Bar graph shows the percentage of hepatocytes with nuclear HMGB1. **F** Serum HMGB1 levels in 9-week-old mice (*n* = 4–7 mice/group). **G** Liver sections were subjected to H&E staining (×200), which showed increased ductular cells in *Atg7−/−* livers and immunostaining for CK19 and Sox9, markers for ductular cells. **H** Liver sections were subjected to Sirius Red staining for fibrosis (×200) and immunostaining for F4/80, a marker of Kupffer’s cells (×200). Data are expressed as the mean ± SEM. *P < 0.05, **P < 0.01, ***P < 0.001, n.s: not significant (*n* = 3).

We thus generated mice deficient in both *Atg7* and *Caspase-11*. We found codeletion of *Caspase-11* did not affect hepatomegaly and liver injury that are characterized by the deletion of Atg7 (Fig. 1A–C). HMGB1 release in autophagy (*Atg7*)-deficient hepatocytes is characterized by the loss of HMGB1 signals in the liver and the elevation of HMGB1 signals in the serum [3]. We found that co-deletion of *Caspase-11* reversed the signal loss of HMGB1 in total liver lysates (Fig. 1D) without affecting p62 accumulation, which was due to defective autophagy function [15]. Consistently, more nuclear retention of HMGB1 was noted in *Atg7/Caspase-11−/−* livers, compared to *Atg7−/−* liver (Fig. 1E). Correspondingly the elevated serum level of HMGB1 was repressed in the doubly deficient mice (Fig. 1F). Functionally, co-deletion of *Caspase-11* caused a dramatic reduction in the expansion of the CK19 and SOX9 positive ductular cell (Fig. 1G), which is known as the ductular reaction and is mediated by released HMGB1 in autophagy-deficient livers [3]. This impact was specific as there was no significant impact on hepatic inflammation or fibrosis (Fig. 1H), which had been shown to be HMGB1-independent [3]. These findings suggest that CASPASE-11 activation is required for active HMGB1 release and functionally for ductular cell expansion in the autophagy-deficient liver.

### Nlrp3 deletion did not prevent the loss of hepatic HMGB1 and expansion of ductular cells

Inflammasome has a role as a sentinel complex in the surveillance of the intracellular environment. It consists of at least two distinct components, pro-CASPASE-1 and a NOD-Like receptor (NLR) molecular or a molecule of the PYHIN family [16]. Most inflammasome protein complexes also contain an adaptor protein, termed apoptosis-associated speck-like protein containing a CARD (ASC), which links pro-CASPASE-1 to the NLR or PYHIN molecules [17]. Human and mouse genome encode more than 20 NLR molecules and several distinct PYHIN molecules. Among these molecules, NLRP3 is the most extensively characterized inflammasome component that has been shown to assemble into functional, caspase-activating complexes [16–18]. Notably, CASPASE-11 can indirectly activate the NLRP3-ASC-CASPASE-1 pathway leading to IL1β/IL-18 processing and release [13, 19].

**Fig. 2:**
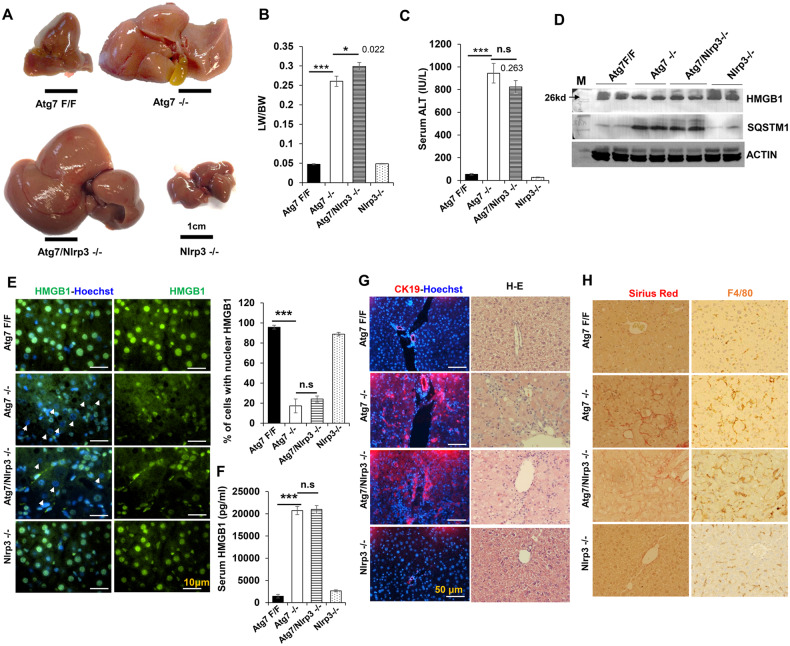
NLRP3 deletion does not block HMGB1 release and ductular cell proliferation in the autophagy-deficient liver. **A** Representative gross morphology of livers of 9-week-old mice of the indicated genotypes. **B**, **C** The LW/BW ratio and the serum ALT levels were determined for 9-week-old mice. **D** Liver lysates from 9-week-old mice were analyzed by immunoblotting. **E** Liver sections were stained for HMGB1. Arrows indicate hepatocytes without nuclear HMGB1. Bar graph shows the percentage of hepatocytes with nuclear HMGB1. **F** Serum HMGB1 levels in 9-week-old mice (*n* = 3–4 mice/group). **G** Liver sections were subjected to H&E staining (×200) and immunostaining for CK19. **H** Liver sections were subjected to Sirius Red staining (×200) and immunostaining for F4/80 (×200). Data are expressed as the mean ± SEM. *P < 0.05, ***P < 0.01, n.s: not significant (*n* = 3).

How individual NLR molecules directly recognize their specific ligands is still not fully understood. PYHIN molecules like AUMS, recognize intracellular double-stranded DNA [16]. NLRP3-mediated inflammasome is activated by exogenous and endogenous danger signals, including DAMP molecules such as ATP, monosodium uric acid crystal, etc. [20]. To determine whether NLRP3 participated in the CASPASE-11 activation mediated HMGB1 release in the autophagy-deficient liver, we generated *Atg7/Nlrp3* doubly deficient mice. Like *Atg7/Caspase-11 −/−* mice, co-deletion of *Nlrp3* did not alter the hepatomegaly and liver injury phenotype of the autophagy-deficient liver (Fig. 2A–C). Further examination of hepatic HMGB1 level showed no inhibition of HMGB1 loss by *Nlrp3* deletion in the autophagy-deficient liver (Fig. 2D, E). Correspondingly the serum level of HMGB1 was still elevated in the doubly deficient mice (Fig. 2F) Neither did *Nlrp3* deletion correct the ductular reaction and hepatic inflammation (Fig. 2G, H). Taken together, these data suggest that NLRP3 may not be important for the CASPASE-11 mediated HMGB1 release and ductular cell expansion in the autophagy-deficient liver.

### Autophagy-deficiency mediated HMGB1 loss from hepatocytes is GASDERMIN D-independent

Activated CASPASE-11 or CASPASE-1 can cleave GASDERMIN D to generate the C terminal domain and the N terminal domain [14, 21]. The N terminal domain of GASDERMIN D can be assembled to form pores on the plasma membrane to allow the passage of various cytoplasmic molecules, such as interleukin-1β (IL-1β), galectin-1 and HMGB1, in macrophages [22, 23]. Eventually, cells with activated CASPASE-11 undergo a lytic form of cell death called pyrotosis.

**Fig. 3:**
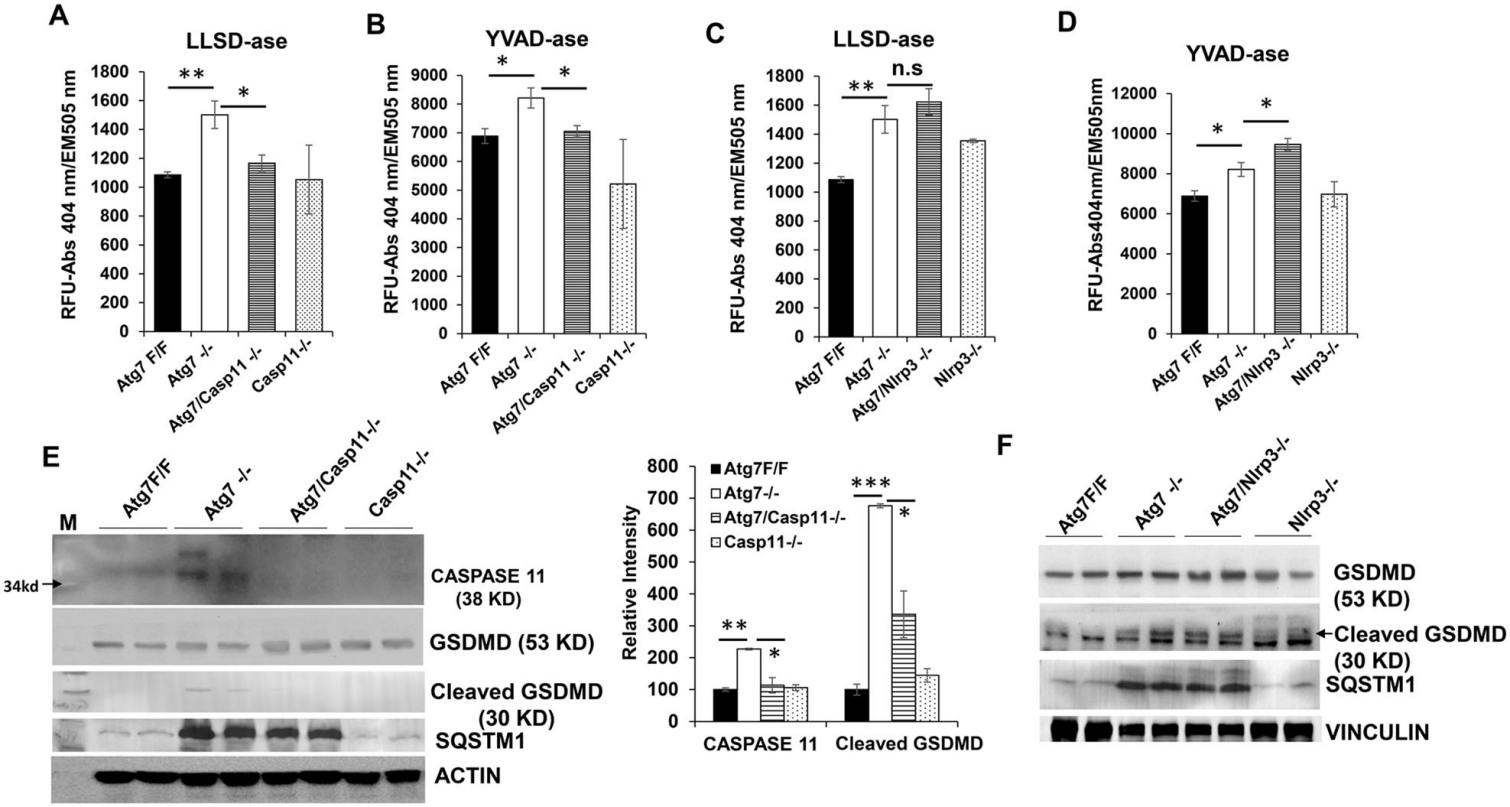
Cleavage of Gasdermin D in autophagy-deficient liver is inhibited by deletion of *Caspase-11*, but not *Nlrp3*. **A**, **C** Liver lysates from 9-week-old mice of the indicated genotypes were analyzed for proteolytic cleavage activity for Ac-LLSD-AFC. **B**, **D** Liver lysates from 9-week-old mice of the indicated genotypes were analyzed for the cleavage activity for Ac-YVAD-AFC. **E**, **F** Liver lysates from 9-week-old mice of the indicated genotypes were analyzed by immunoblotting. The GASDERMIN D (GSDMD) fragment, indicated by the arrow, was detected using an antibody specific to the N-terminal fragment. Bar graph shows the relative intensity of caspase-11 and cleaved GSDMD. Data are expressed as the mean ± SEM. *P < 0.05, **P < 0.01, n.s: not significant (*n* = 3).

To determine the proteolytic activity against GASDERMIN D in the autophagy-deficient livers, we used a peptide with the CASPASE-1 and CASPASE-11 cleavage site derived from GASDERMIN D, LLSD [24], coupled with the fluorochrome AFC. The cytosolic LLSD-AFC cleavage activity, or LLSDase activity, was elevated in *Atg7−/−* livers, but was abolished by co-deletion of *Caspase-11* (Fig. 3A). The proteolytic activity against the peptide YVAD-AFC, which represents the sequence of the CASPASE-1 cleavage site in IL-1β, was also elevated in autophagy deficient livers as previously described [3], and was also abolished by *Capase-11* co-deletion (Fig. 3B). These data suggest that CASPASE-11 is critical for not only GASDERMIN D cleavage also the activity of CASPASE-1. In contrast, neither the LLSDase activity (Fig. 3C) nor the YVADase activity (Fig. 3D) was inhibited by the deletion of *Nlrp3* in the autophagy-deficient liver. Consistent with the elevated LLSDase activity, cleaved GASDERMIN D fragment was detected in *Atg7−/−* livers in a *Caspase-11-*, but not *Nlrp3-*, dependent manner (Fig. 3E, F). These observations are consistent with the finding that CASPASE-11 but not NLRP3, is necessary for HMGB1 release in the autophagy-deficient liver (Figs. 1, 2).

**Fig. 4:**
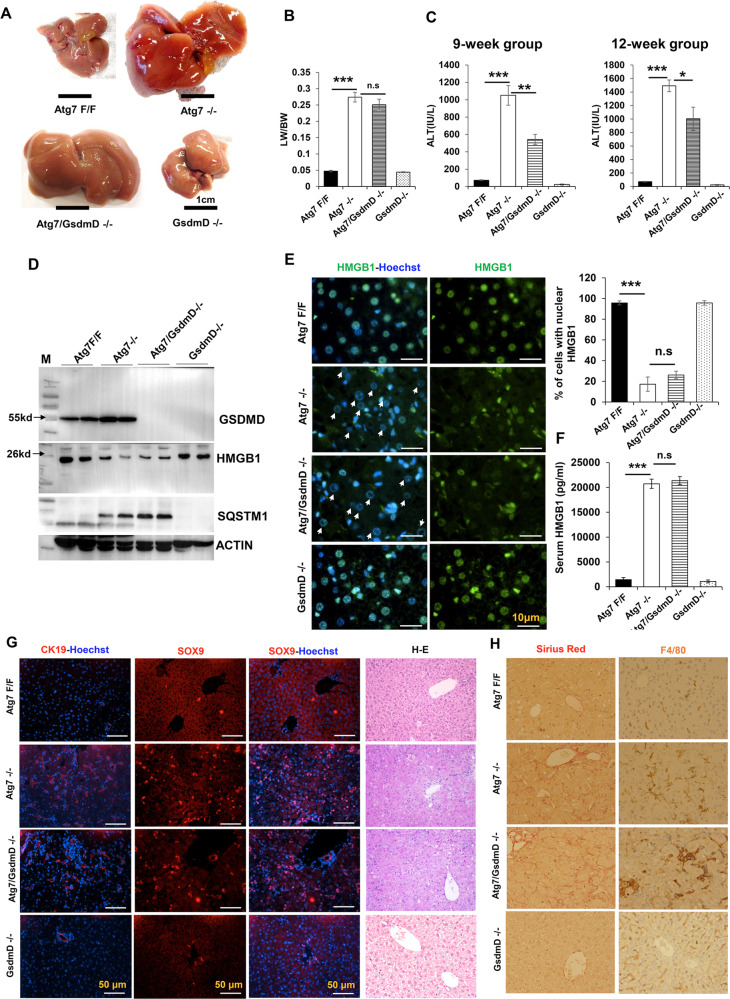
*Gasdermin D* deletion does not prevent hepatic HMGB1 release and ductular reaction. **A** Representative gross morphology of livers of 9-week-old mice of the designated genotypes. **B**, **C** The LW/BW ratio and the serum ALT levels were determined for 9-week-old and 12-week-old mice. **D** Liver lysates from 9-week-old mice were analyzed by immunoblotting. **E** Liver sections were stained for HMGB1. Arrows indicate hepatocytes without nuclear HMGB1. Bar graph shows the percentage of hepatocytes with nuclear HMGB1. **F** Serum HMGB1 levels in 9-week-old mice (*n* = 3–4 mice/group). **G** Liver sections were subjected to H&E staining (×200) or immunostaining for CK19 and SOX9. **H** Liver sections were subjected to Sirius Red staining (×200) or immunostaining for F4/80 (×200). Data are expressed as the mean ± SEM. *P < 0.05, **P < 0.01, **P < 0.001, n.s: not significant (*n* = 3).

We next examined whether GASDERMIN D might be directly involved in the HMGB1 release using *Atg7/Gasdermin D* doubly deficient mice. *Gasdermin D* deletion did not affect hepatomegaly (Fig. 4A, B), but partially protected against liver injury as measured by the serum level of ALT (Fig. 4C). Surprisingly, co-deletion of *Gasdermin D* did not repress HMGB1 release in autophagy-deficient livers (Fig. 4D–F), nor the ductular reaction (Fig. 4G), nor hepatic inflammation and fibrosis (Fig. 4H). These results thus indicate that while CASPASE-11 is activated and can cleave GASDERMIN D, the latter is dispensable for HMGB1 release by autophagy-deficient hepatocytes and the downstream effect of the released HMGB1.

### *Caspase-11* mRNA expression can be regulated by NRF2

We previously reported that autophagy-deficient livers had an increased transcriptional level of *Caspase-11* [3]. CASPASE-11 can be auto-activated at a high expression level and can in turn active CASPASE-1 [25, 26]. Thus, transcriptional upregulation of *Caspase-11* may contribute to an increased CASPASE-11 and CASPASE-1 activities in autophagy-deficient livers. Since NRF2 is persistently activated in autophagy deficient livers and genetic co-deletion of *Nrf2* inhibits CASPASE-11 upregulation [3], we examined whether NRF2 could be directly responsible for the transcriptional upregulation of *Caspase-11*.

**Fig. 5:**
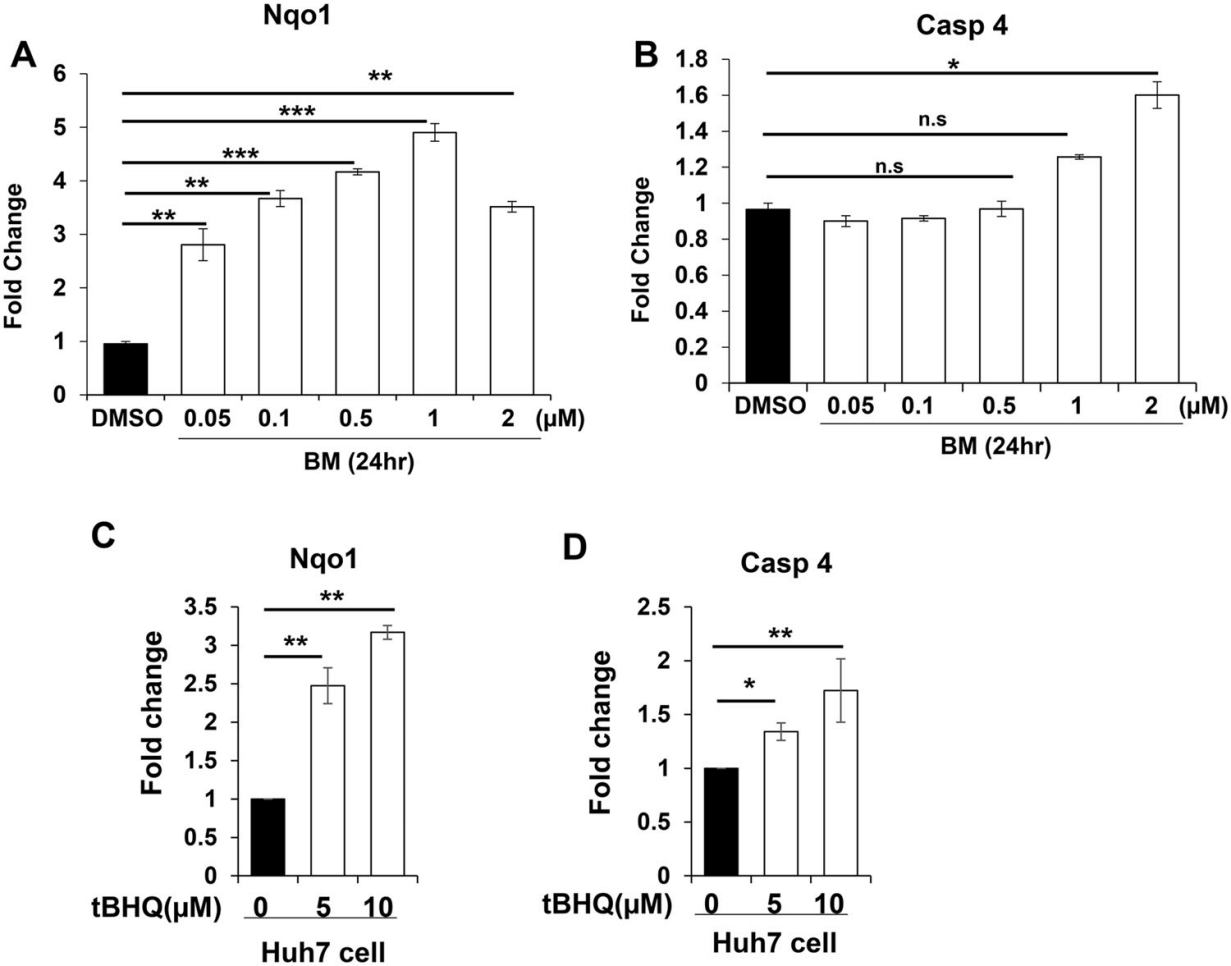
NRF2 activators induce mRNA expression of Caspase-4 in the human hepatic cell line. **A**, **B** Treatment with BM for 24 h induced *Nqo1* (**A**), and *Caspase-4* (**B**) expression in the human liver cancer cell line Huh7. **C**, **D** Treatment with tBHQ for 24 h induced *Nqo1* (**C**), and *Caspase-4* (**D**) expression in Huh7. mRNA expression levels were quantified by RT-PCR and normalized to that of β-actin. *P < 0.05, **P < 0.01, **P < 0.001, n.s: not significant (*n* = 3).

We first treated the Huh7 human hepatic cell line with different doses of Bardoxolone methyl (BM). BM is a class of synthetic triterpenoids that can pharmacologically activate NRF2 in vitro and in vivo [27]. Indeed, the transcription of *Nqo1*, a bona fide target gene of NRF2, was stimulated by BM in a dose-dependent manner (Fig. 5A). Likewise, the transcription of *Caspase-4*, the human counterpart of mouse *Caspase-11*, was significantly upregulated by BM, although at a higher dose (Fig. 5B). Similar elevation of *Nqo1* and *Caspase-11* transcription were observed in Huh7 cells treated with another NRF2 activator *tert*-butylhydroquinone (tBHQ) (Fig. 5C, D). These data support that *Caspase-11/Caspase-4* can be a NRF2 transcription target.

**Fig. 6:**
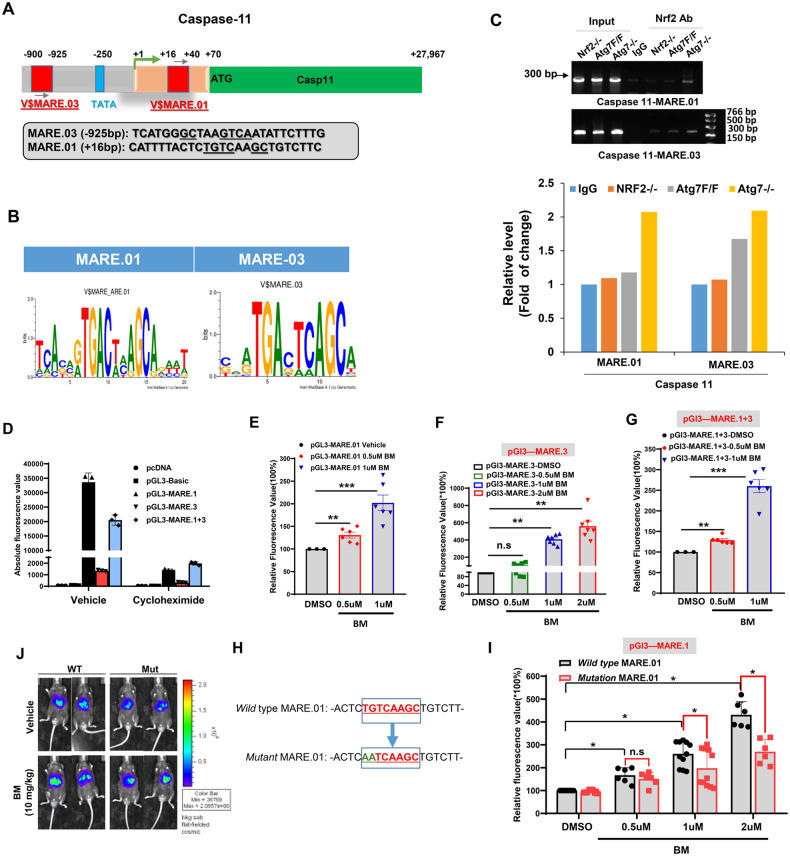
NRF2 transcriptionally upregulates *Caspase-11* expression. **A** Endogenous NRF2 binding regions-MARE.01 and MARE.03 in mouse *Caspase-11* promotor. Potential NRF2 binding sites are highlighted in red block and the DNA sequences are shown in the box. The underlined nucleotides are the core sequences of the MARE.01 and MARE.03. **B** The sequence motif in MARE.01 and MARE.03 as identified by the software Mat Inspector. **C** Chromatins prepared from livers of indicated genotypes were subjected to CHIP analysis using a specific anti-NRF2 antibody. Specific bands were detected for the MARE.01 and MARE.03 sequences in the *Caspase-11* promoter from immunoprecipitated of the *Atg7−/−* livers. **D** Luciferase reporter assay was conducted with 293-T cells transfected with different plasmid constructs and treated with BM (2 µM) for 24 h before luciferase measurement. In a parallel group, the protein synthesis inhibitor cycloheximide (100 µg/ml) was added that suppressed the expression of the luciferase. The renilla luminescence was used to normalize the firefly luminescence. **E**−**G** Dose-dependent effect of NRF2 activation on the activity of luciferase under the control of MARE.01 (**E**), MARE.03 (**F**), or the combination of the two (**G**). **H** Site-directed mutagenesis of the MARE.01 core sequence. **I** 293-T cells were transfected with reporter vectors with mutated or wild-type MARE.01 sequence, followed by treatment with different concentrations of BM for 24 h before the luciferase activity was measured. **J** Mice were intravenously given luciferase reporter constructs with mutated or wild-type MARE.01 sequence. They were treated with BM (10 mg/kg) for 24 h before the in vivo luciferase activity was measured. Data are expressed as the mean ± SEM. *P < 0.05, **P < 0.01, **P < 0.001, n.s: not significant (*n* = 3).

NRF2 is a transcription factor that normally responds to oxidative stress by binding to antioxidative response element (ARE) present in the promoter region of its downstream target genes such as *Nqo1* [28]. We therefore examined the promoter region of *Caspase-11* for the presence of mouse antioxidative response element (MARE) regulatory element (Fig. 6A). We performed in silico promoter analysis of *Caspase-11* for the presence of conserved MARE elements using MatInspector (https://www.genomatix.de) and JASPER [29]. Both MatInspector and JASPER identified two putative MAREs where NRF2 could bind. These two sites are located from −900 to −925 bp upstream of the transcription start sites (TSS) (MARE.03) and +16 to +40 downstream of TSS (MARE.01) (Fig. 6A, B).

To determine whether NRF2 can directly bind to the putative MAREs in the *Caspase-11* promoter, a CHIP assay was performed with the chromatin prepared from the *Atg7 F/F, Atg7 −/−* and *Nrf2 −/−* liver samples and with oligonucleotides flanking MARE.03 or MARE.01 following our previously established protocol [30]. As shown in Fig. 6C, a specific PCR product corresponding to the MARE.01 or MARE.03 region could be amplified from *Atg7−/−* liver chromatins immunoprecipitated with an anti-NRF2 antibody. In contrast, few PCR products were detected from chromatins prepared from the floxed *Atg7* or *Nrf2 −/−* livers. These findings suggest that NRF2 can be recruited and bound to the MARE.03 and MARE.01 sites in the proximal *Caspase-11* promotor in the *Atg7−/−* liver (Fig. 6C). This data suggests that NRF2 directly binds to the evolutionarily conserved MARE sequences at *Caspase-11* promotor.

To determine whether the putative MARE regulatory sequences were involved in the transcriptional regulation of *Caspase-11* expression, we transfected a luciferase reporter construct containing the MARE sequence into 293 T cells, followed by the treatment with BM to activate NRF2. The results showed that both MARE.01 and MARE.03 could mediate the NRF2-activated luciferase activity (Fig. 6D). MARE.01 sequence demonstrated a higher inducibility than the MARE.03 sequence. Moreover, luciferase activities were increased in a dose-dependent manner with the amount of BM used to induce NRF2 activity BM (Fig. 6E–G). To determine the dependence of the luciferase activity on the conserved NRF2 binding nucleotide sequences, we mutated the core region of MARE.01 (Fig. 6H) and found that the mutation led to significantly lower luciferase activities upon the stimulation of a higher dose of BM (Fig. 6I).

Finally, to assess if NRF2 plays a functional role in *Caspase-11* expression in vivo, we injected the luciferase reporter construct with wild type or mutated MARE.01 sequence to mice via tail vein, which led to the enrichment of the construct. in the liver. The mice were then given BM or vehicle control 72 h later (Fig. 6J). There was a low basal expression of the luciferase in the livers of the vehicle-treated mice, which was lower in mice receiving the reporter with the mutated MARE.01 sequence. Pharmacological activation of NRF2 by BM significantly increased the luciferase expression in mice receiving the reporter with the wild type of MARE.01 sequence. In mice given the mutant pGL3-MARE.01-luciferase construct the elevation of luciferase activity was much limited upon BM treatment.

These results indicate that NRF2 can bind to specific MARE sequences present in the promoter region of *Caspase-11* and drive transcriptional upregulation of a reporter gene in vitro and in vivo. Taken together, all the observations support the notion that NRF2 can transcriptionally activate *Caspase-11*, and an increased level of CASPASE-11 leads to auto-activation that is responsible for HMGB1 release.

## Discussion

**Fig. 7:**
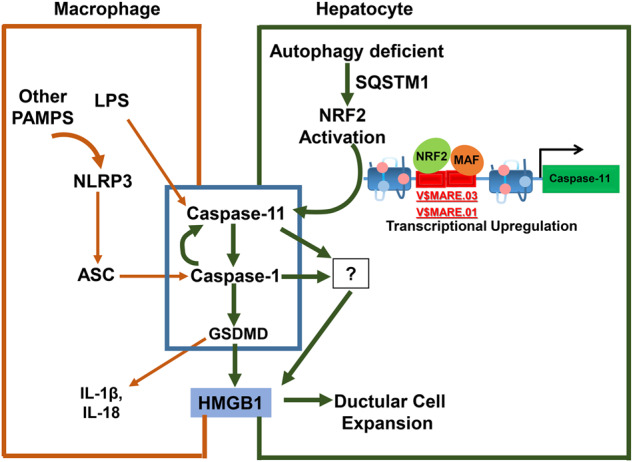
NRF2-mediated transcriptional activation of *Caspase-11* and HMGB1 release from autophagy-deficient hepatocytes. LPS stimulated macrophages cause HMGB1 active release through activation of NLRP3, Capase-11 inflammasomes leading to activation of Caspase-1 and subsequent cleavage of Gasdermin D(GSDMD). Active release of HMGB1 by autophagy-deficient hepatocytes is mediated by transcriptional activation of Caspase-11 by NRF2, in which NLRP3 and GSDMD can be dispensed.

In this study, we sought to determine the mechanism involved in the active release of HMGB1 by autophagy-deficient hepatocytes. This area of investigation is important because extracellular HMGB1 is critical for the pathogenesis in autophagy-deficiency livers by promoting progenitor cell proliferation (ductular reaction) and tumorigenesis [3]. HMGB1 release and pathological effects of extracellular HMGB1 have also been demonstrated in many other types of liver diseases including alcoholic and non-alcoholic fatty liver diseases, liver injury caused by 3,5-diethoxycarbonyl-1,4-dihydrocollidine(DDC) diet or Choline-deficient, Ethionine-supplemented (CDE) diet, and liver cancers caused by carcinogens [3, 4, 6]. The present study fills the gaps in our understanding of the molecular mechanism of HMGB1 release by autophagy deficient hepatocytes, which is different in many aspects from the similar process in LPS-stimulated macrophages [31–33](Fig. 7).

### CASPASE-11 is a novel NRF2 transcriptional target

NRF2 is a member of the cap ‘n’ collar (CNC) subfamily of the basic region leucine zipper (bZIP) transcription factor [28]. It can activate many genes via binding to antioxidant response element (ARE) commonly present in the promoter region of many antioxidative stress response genes such as *Nqo, Gstm1*, and *Ho-1* [28]. Induction of these enzymes leads to increased detoxification of oxidants and electrophiles and protects cells against oxidative stress. NRF2 is overly activated in autophagy-deficient hepatocytes, which is associated with the liver pathology [3, 15]. We have also shown that such activated NRF2 can trigger the inflammasome [3, 15]. The present study provides the evidence that NRF2 can accomplish this function by transcriptionally upregulate *Caspase-11* expression. We have earlier reported [3] that the mRNA expression levels of Casp11 is elevated in the *Atg7−/−* liver in an NRF2-dependent manner. Moreover, the appearance of the activated subunit of Caspase-11 was detected in *Atg7−/−* liver, but not in the Atg7Nrf2−/− livers. Furthermore the LLSDase activity (mainly Caspase-11) were also elevated in *Atg7−/−* liver and were abolished by co-deletion of NRF2 in the autophagy-deficient liver. These data clearly suggest that NRF2 controls Caspase-11 in liver. We have now specifically shown the presence of active NRF2 binding sites in the *Caspase-11* proximal promoter region. CHIP analysis confirms the binding of NRF2 to these sites in a way driven by autophagy deficiency. Moreover, isolated sequences from these binding sites can drive the expression of luciferase reporter in vitro and in vivo. Since NRF2 can be activated in many cases other than in autophagy deficient hepatocytes, the finding that NRF2 can activate inflammasomes by transcriptionally upregulate CASPASE-11 provides a potential mechanistic explanation in other cases where NRF2 is engaged in inflammasome activation [34].

In contrast, upregulation of *Caspase-11* in macrophage is driven by stimulators such as LPS and by the surge in the intracellular level of cAMP stimulated by the stress hormone and neurotransmitter [35]. CASPASE-11 can be activated through self-oligomerization that is driven by increased levels of this molecular and/or direct binding of cytosolic LPS. The lipid A moiety of cytosolic LPS can bind to the caspase activation and recruitment domain (CARD) of CASPASE-11 [36]. We had reported that the autophagy-deficient liver has altered gut microbiota due to hepatic cholestasis [37, 38]. Alteration of gut microbiota in the autophagy-deficient liver may impact the hepatic LPS level. However, we have not found any significant elevation of bacterial 16 S rRNA in the autophagy-deficient livers (data not shown). Hence LPS binding to CASPASE-11 may not occur or may not be sufficient to activate CASPASE-11. While this effect could not be completely ruled out, it is more likely that the upregulation of CASPASE-11 leads to self-oligomerization and activation [25, 26, 39, 40].

Additionally, NLRP3 is shown to participate in an amplification loop of CASPASE-1 and CASPASE-11 activation, and the release of IL-1β by LPS-stimulated macrophages [41]. However, the present study indicates that NLRP3 is dispensable for HMGB1 release by autophagy-deficient hepatocytes. This finding suggests that CASPASE-11 alone could be sufficient to induce the inflammasome-dependent release of HMGB1 in hepatocytes.

### Extracellular release of HMGB1 by a GASDERMIN D-independent mechanism

The active secretion of HMGB1 required at least two steps. First, HMGB1 translocates from the nucleus to cytosol, and second, HMGB1, a leaderless protein, is released into extracellular space crossing the plasma membrane. While the nuclear translocation of HMGB1 is known to be regulated by the acetylation of the molecule [33], its active release or secretion to the extracellular space, like several other leaderless molecules, IL-1β, and IL-18 is shown to involve GASDERMIN D, which is cleaved and activated by CASPASE-1 and/or CASPASE-11, in macrophages [14, 21]. The N-terminal fragment of GASDERMIN D forms pores on the cytoplasmic membranes, which are about 10- to 14 nm in diameter and can be responsible for the active release of the leaderless molecules and for the subsequence cell death through pyroptosis [24, 42]. Pore formation in the cell membrane is a conserved strategy for the release of intracellular factors. Pore-forming proteins such as GASDERMIN D can selectively transport factors between cytosol and extracellular space [43].

Our study indicates that GASDERMIN D is dispensable for the active release of HMGB1 by autophagy-deficient hepatocytes even though the cleavage of GASDERMIN D occurs. There are likely other consequences of GASDERMIN D cleavage or pore formation since deletion of GASDEMIN D resulted in a reduction of the blood ALT levels, suggesting a reduction of hepatocyte pyroptosis. However, more importantly, this result suggests there are other possible mechanisms contributing to HMGB1 release.

Another possible pore-forming protein for HMGB1 release by the autophagy-deficient liver could be mixed lineage kinase domain-like (MLKL). MLKL is a cytosolic, monomeric pseud-kinase with unknown function in healthy cells [44]. MLKL is activated via phosphorylation by receptor-interacting protein 3 (RIP3). MLKL activation triggers oligomerization and association with the inner leaflet of the plasma membrane, which eventually leads to membrane pore formation [44]. In addition, leaderless proteins may be released through extracellular vehicles (EVs) [45]. Interestingly MLKL could also facilitate endosomal trafficking and EVs generation and release of intracellular proteins [46]. The role of MLKL, EVs and other possible mechanisms in CASPASE-11-depdendent but GASDERMIN D-independent release of HMGB1 by the autophagy-deficient hepatocytes will need to be investigated in future studies.

In summary, the present study had defined CASPAES-11 as a novel transcriptional target of NRF2 and revealed how NRF2 can participate in the activation of inflammasome. Furthermore, we reveal the presence of a GASDERMIN D-independent pathway downstream of the inflammasome for the active release of HMGB1 and possibly other leaderless molecules in non-immune epithelial cells.

## Materials/Subjects and Methods

### Animal models

Wild-type C57BL/6 mice and mice with floxed *Atg7* (*Atg7F*/*F*) [37], with hepatic deletion of *Atg7* (*Atg7−/−* mice) [15], with constitutively activated *Nrf2* (*CaNRF2*) [47], or with *Alb-Cre* expression [48] (The Jackson Laboratory, Cat#003574) have been described previously. These mice were further crossed with mice deficient in *Caspase-11*(The Jackson Laboratory, Cat#024698), in *Nlrp3* (The Jackson Laboratory, Cat#021302), or in *Gasdermin D* [14] to generate *Atg7/Caspase11 −/−* or *Atg7/Nlrp3 −/−*, or *Atg7/GsdmD −/−* doubly deficient mice as previously described [3]. Both male and female mice were used at the ages between 6-12 weeks. Mice were housed in a pathogen-free facility and were maintained on a 12-hour light/12-hour dark cycle with free access to food and water. The Institutional Animal Care and Use Committee (IACUC) of Tulane University and Indiana University approved all animal studies.

### Reagents and cell lines

Antibodies and primers used in this study are listed in Table S1 and Table S2, respectively. HEK293 and Huh7 cell lines were purchased from the American Type Culture Collection (ATCC). Cells were cultured in Dulbecco’s modified Eagle’s medium (DMEM) supplemented with 10% fetal bovine serum, streptomycin (100 µg/mL) and penicillin (100 U/mL) (Invitrogen, USA). Cells were maintained in a 5% CO_2_ atmosphere at 37 °C.

### Luciferase reporter assay

DNA element containing NRF2 binding motifs-ARE.01 and ARE.3 in the promotor region of *Caspase-11* was identified by MatInspector analysis. ARE.1 and ARE.3 containing sequences were cloned into pGL3-firefly luciferase expression vector (Promega) as the reporter plasmid. Renilla Luciferase (R-Luc) was used to normalize the transfection efficiency of the reporter plasmid. The total amount of plasmids was kept constant by adding an equal dose of corresponding empty vectors. Plasmids were transfected into HEK293 cells using Lipofectamine 2000 (Thermo Fisher) according to the manufacturer’s instructions. HEK293 cells were then treated with different concentrations of an NRF2 activator bardoxolone methyl (Cat No 6646, Tocris). A dual-luciferase assay kit (E1980, Promega) was used to measure both firefly and *Renilla* luciferase activities according to the manufacturer’s instructions. Results were quantified as the ratio of the firefly/*Renilla* luciferase activities and were representative of at least two independent experiments.

For in vivo luciferase assay, ARE.1 containing pGL3-firefly luciferase expression vector was intravenously administered to wild type C57BL/6 mice via hydrodynamic method. Mice were then intraperitoneally injected with 10 mg/kg body weight of bardoxolone methyl. Twenty-four hours later, mice were given intraperitoneally 150 mg/kg of D-luciferin in PBS and the in vivo luciferase activity was imaged using an IVIS 2000 imaging system. Photon flux was quantified using the Living Image 3.0 software.

### Chromatin Immunoprecipitation (CHIP) assay

CHIP was performed using established protocols as previously described [37]. Briefly, liver tissue was fixed and cross linked with 1% formaldehyde and then quenched with 0.125 M glycine (G36050, RPI). The nucleus was extracted and sonicated to yield genomic DNA fragments of 300~800 bp in length. Aliquot of chromatin was immunopurified using an anti-NRF2 antibody suitable for CHIP assay or a control rabbit IgG. Dynabeads Protein A/G beads (10009D, Thermo Fisher Scientific) was used to pull down chromatin-antibody complexes. The immunoprecipitated DNA fragments were purified by phenol-chloroform extraction and subjected to qPCR with specific primers.

### Measurement of caspase activity

Liver tissues were lysed in the caspase activity buffer [3], and the lysate (20–40 μg) were incubated with 80 μm of Ac-LLSD-AFC or Ac-YVAD-AFC for 1–16 h. at 37 °C for measuring the activity of CASPASE-11 or CASPASE-1, respectively, with a fluorescence spectrometer (Tecan Infinite M200 Pro) at the excitation/emission wavelength length of 400 nm/505 nm. Fluorescence readings were normalized to the background and standardized to the amount of protein analyzed.

### Immunoblotting analysis

Immunoblotting assay was conducted as previously described [3]. Briefly, liver tissues were lysed in RIPA buffer containing protease inhibitor cocktail. Total liver lysates of approximately 40 µg per sample were subjected to SDS-PAGE, followed by immunoblotting. The results were digitally acquired using Bio-Rad ChemiDoc Image System and densitometry was performed using the Image-Lab (BioRad) software.

### Enzyme-linked immunosorbent assay (ELISA)

The levels of Serum HMGB1 protein were determined using the mouse HMGB1 ELISA kit (LS Bio, Cat# LS-F4040-1) in accordance with the manufacturer’s instructions.

### Real-time quantitative PCR analysis

Total RNA was extracted from liver tissue samples using a GeneTET RNA Purification Kit (Thermo Scientific) according to the manufacturer’s protocol. cDNA was prepared with 1 μg total RNA using OligodT primers and M-MLV Reverse Transcriptase Enzyme System (Life Technologies, Thermo Fisher Scientific). Quantitative real-time Polymerase chain reaction (qRT-PCR) was performed using SYBR Green Master Mixes on a 7500 FAST Real-Time PCR System (Life Technologies–Applied Biosystems, Thermo Fisher Scientific). Gene expression was quantified using the 2–${\Delta}{\Delta}$Ct method and normalized to the housekeeping gene *actin or Gapdh*.

### General Histology, Immunostaining, and Immunohistochemistry

Mouse liver tissues were fixed in 10% neutral buffered formalin, embedded in paraffin. A separate portion of mouse liver was fixed with 4% paraformaldehyde and embedded with Tissue-Tek optimum cutting temperature compound (OCT) under frozen condition. The paraffin sections (4 μm) were prepared for histological staining. For immunofluorescence staining, deparaffinized or frozen sections were subjected to antigen retrieval in citrate buffer (pH6.0). Slides were permeabilized and blocked with 5% goat or donkey serum in PBS containing 0.1% Triton X (PBS-Tx) and glycine for 1 h at room temperature and then incubated overnight at 4 °C with primary antibodies diluted in PBS. Sections were washed in PBS-Tx, following by incubation with fluorochrome-conjugated secondary antibodies. Hoechst 33342 (1 μg/ml) was used for the staining of the nucleus. Histology images were obtained using a Nikon Eclipse E200 microscope equipped with a SPOT RT Slider color digital camera (Diagnostic Instruments), and immunofluorescence images were obtained using a Nikon Eclipse TE 200 epi-immunofluorescence microscope and the companion NIS-Elements AR3.2 software.

### Statistical analysis

Statistical analysis was performed using SigmaStat 3.5 (Systat Software). Data were analyzed from at least three independent experiments or samples. Data are shown as means ± SEM. Student’s *t*-test or one-way analysis of variance test were employed and *p* < 0.05 was considered to be statistically significant.

## Supplementary information

Table S1-Antibodies

Table S2- QPCR Primer list

## Data Availability

All data generated or analyzed during this study are included in this published article and its supplementary information files.
