# Supplementary material for: NRF2 transcriptionally regulates Caspase-11 expression to activate HMGB1 release by Autophagy-deficient hepatocytes
Source: Cell Death Discov. 2023 Jul 28;9:270. doi: 10.1038/s41420-023-01495-x (PMC10382497; doi:10.1038/s41420-023-01495-x)
Supplement: Supplementary file 1 — Table S1-Antibodies [file 41420_2023_1495_MOESM1_ESM.pdf]

Table S1.Antibodies for immnostaining and westernblot

Antibody/Species	Source/Catalogue Number/Dilution
Actin/Mouse	Sigma/5441/1:5000
Alexa-488-antiRabbit	InVitrogen/A-11034/1:500
Caspase-11/Rat	Cell Signal/14340/1:1000
CK19/TROMA-III/Rat	Developmental Studies Hybridoma Bank(DSHB)/1DB-001-0000868971/1:200
Cy3-Anti Rat	Jackson ImmunoResearch Laboratories Inc/712-165-150/1:500
F4/80	Bio-Rad/MCA497G/1:100
Gapdh/Mouse	Novus/NB300-21/1:3000
GasderminD-cleaved/Rabbit	Cell siganling/10137S/1:1000
GasderminD-Full length/Rabbit	Abcam/ab219800/1:1000
Hmgb1/Rabbit	Abcam/ab18256/1:1000
HRP-labeled Mouse secondary antibody	Jackson ImmunoResearch Laboratories Inc/115-035-062/1:5000
HRP-labeled Rabbit secondary antibody	Jackson ImmunoResearch Laboratories Inc/111-035-045/1:5000
p62/SQSTM1/Mouse	Abnova/H00008878-M01/1:1000
Sox9/Rabbit	EMB Millipore/AB5535/1:1000
Vinculin/Rabbit	Novus Biologicals/NBP2-20859/1:1000
