# Supplementary material for: NRF2 transcriptionally regulates Caspase-11 expression to activate HMGB1 release by Autophagy-deficient hepatocytes
Source: Cell Death Discov. 2023 Jul 28;9:270. doi: 10.1038/s41420-023-01495-x (PMC10382497; doi:10.1038/s41420-023-01495-x)
Supplement: Supplementary file 2 — Table S2- QPCR Primer list [file 41420_2023_1495_MOESM2_ESM.pdf]

Table S2.Primers used for RTPCR, QPCR and CHIP-qPCR experiment

Gene name	Forward primer	Reverse Primer
**Mouse**		
Caspase-11-MARE.01	5'-CCCACAGCAGTTGATACTTTCT-3'	5'-ATTCTAGGACTCAAGTCAAAGCC-3'
Caspase-11-MARE.03	5'-CTTATTACTCTGGAAGCCTGGAC-3'	5'-CCATCTCCTCAGTGTCATACTTATC-3'
**Human**		
Nqo1	5'-GCACTGATCGTACTGGCTCA-3'	5'-CATGGCATAGAGGTCCGACT-3'
Caspase 4	5'-GAATCTGACAGCCAGGGATATG-3'	5'-CCATGAGACATGAGTACCAAGAA-3'
Actin	5'-GGCATCCTCACCCTGAAGTA-3'	5'-AGGTGTGGTGCCAGATTTTC-3'
Gapdh	5'-CAATGACCCCTTCATTGACC-3'	5'-GACAAGCTTCCCGTTCTCAG-3'
